# Pilot Study Examining the Influence of Potassium Bicarbonate Supplementation on Nitrogen Balance and Whole-Body Ammonia and Urea Turnover Following Short-Term Energy Restriction in Older Men

**DOI:** 10.3390/nu10050624

**Published:** 2018-05-16

**Authors:** Lee M. Margolis, Lisa Ceglia, Donato A. Rivas, Bess Dawson-Hughes, Roger A. Fielding

**Affiliations:** 1 Nutrition, Exercise, Physiology, and Sarcopenia Laboratory, United States Department of Agriculture Jean Mayer Human Nutrition Research Center on Aging, Tufts University, Boston, MA 02111, USA; lee.margolis@tufts.edu (L.M.M.); donato.rivas@tufts.edu (D.A.R.); 2 Bone Metabolism Laboratory, United States Department of Agriculture Jean Mayer Human Nutrition Research Center on Aging, Tufts University, Boston, MA 02111, USA; lisa.ceglia@tufts.edu (L.C.); bess.dawson-hughes@tufts.edu (B.D.-H.); 3 Division of Endocrinology, Diabetes, and Metabolism, Tufts Medical Center, Boston, MA 02111, USA

**Keywords:** weight loss, aging, alkaline supplement, acid-base

## Abstract

With aging there is a chronic low-grade metabolic-acidosis that may exacerbate negative protein balance during weight loss. The objective of this randomized pilot study was to assess the impact of 90 mmol∙day^−1^ potassium bicarbonate (KHCO_3_) versus a placebo (PLA) on 24-h urinary net acid excretion (NAE), nitrogen balance (NBAL), and whole-body ammonia and urea turnover following short-term diet-induced weight loss. Sixteen (KHCO_3_; *n* = 8, PLA; *n* = 8) older (64 ± 4 years) overweight (BMI: 28.5 ± 2.1 kg∙day^−1^) men completed a 35-day controlled feeding study, with a 7-day weight-maintenance phase followed by a 28-day 30% energy-restriction phase. KHCO_3_ or PLA supplementation began during energy restriction. NAE, NBAL, and whole-body ammonia and urea turnover (^15^N-glycine) were measured at the end of the weight-maintenance and energy-restriction phases. Following energy restriction, NAE was −9.8 ± 27.8 mmol∙day^−1^ in KHCO_3_ and 43.9 ± 27.8 mmol∙day^−1^ in PLA (*p* < 0.05). No significant group or time differences were observed in NBAL or ammonia and urea turnover. Ammonia synthesis and breakdown tended (*p* = 0.09) to be higher in KHCO_3_ vs. PLA following energy restriction, and NAE was inversely associated (*r* = −0.522; *p* < 0.05) with urea synthesis in all subjects. This pilot study suggests some benefit may exist with KHCO_3_ supplementation following energy restriction as lower NAE indicated higher urea synthesis.

## 1. Introduction

The increased prevalence of obesity in older adults is a major health and economic concern, increasing the risk of developing comorbidities, such as type 2 diabetes and cardiovascular disease, and raising medical costs with per capita spending being greatest among Medicare recipients [1,2,3]. Despite evidence of modest weight loss (5–10% of body weight) improving body composition (e.g., reductions in fat mass) and metabolic and cardiovascular parameters [4,5,6], controversy exists regarding weight loss for older adults due to unintentional losses of skeletal muscle mass [7]. As older individuals may already have compromised skeletal muscle mass (i.e., sarcopenia) [8], further declines with weight loss may exacerbate reductions in physical function and increase frailty [7]. Thus, weight loss treatment approaches for the overweight and obese elderly require further studies to elucidate the mechanism associated with attenuating losses of skeletal muscle mass [9].

Aging results in a chronic low-grade metabolic acidosis [10] that may be detrimental to protein metabolism [11], promoting negative protein balance with resulting declines in muscle mass. Provision of an alkalizing agent to correct the low-grade metabolic acidosis may alleviate decrements to protein metabolism and aid in maintaining skeletal muscle mass with aging. During periods of weight maintenance, high consumption of alkaline rich foods, such as fruit and vegetables, has been observed to favor the preservation of muscle mass in older men and women [12]. This attenuated decline in muscle mass with aging may be due to reductions in utilization of nitrogen as a buffer. Metabolic acidosis results in a greater reliance of nitrogen-containing ammonia (NH_3_) to buffer elevated H^+^, forming ammonium (NH_4_^+^). Greater reliance of nitrogen as a buffer may result in negative nitrogen balance, an indicator of muscle loss [13]. Several studies [14,15,16,17] have reported that supplementation with potassium bicarbonate (KHCO_3_), an alkalinizing salt, reduces urinary nitrogen excretion. This reduced nitrogen excretion may improve skeletal muscle integrity and minimize muscle mass losses during weight loss by maintaining nitrogen and whole-body protein balance. Whether reduction in nitrogen excretion with KHCO_3_ supplementation improves nitrogen balance and maintains whole-body protein turnover during energy restriction and weight loss is unknown.

The objective of this pilot study was to examine whether consuming a KHCO_3_ versus a matched placebo supplement alters whole-body protein turnover and nitrogen balance in response to 28 days of a 30% energy restriction in older-age (60–75 years) men. To the best of our knowledge, this was the first study of its kind to assess the effects of KHCO_3_ supplementation on protein metabolism during energy restriction. As such, the primary aim of this pilot study was to determine alterations in ammonia and urea turnover and nitrogen balance during energy restriction with or without KHCO_3_ supplementation to establish an effect size to be utilized for potential future larger randomized trials.

## 2. Materials and Methods

### 2.1. Participants

Participants were sedentary (<2 exercise sessions per week), overweight/obese (25–35 kg·m^2^) men between 60 and 75 years of age, willing to consume only foods and beverages provided by the Human Nutrition Research Center on Aging (HNRCA) metabolic kitchen, abstain from alcohol, tobacco, and dietary supplement use, and maintain their level of physical activity. Participants were in good health and free of any chronic disease as determined by medical evaluation. This study was approved by the Tufts University Health Sciences Campus Institutional Review Board, with documented informed written consent obtained from all participants prior to data collection.

### 2.2. Study Design

In this randomized, placebo-controlled study, participants were randomly assigned to receive 90 mmol∙day^−1^ KHCO_3_ (*n* = 8) or a matched placebo (PLA; *n* = 8) during 28 days of a 30% energy restriction. Participants were placed on a eucaloric (weight maintenance) diet for the first 7 days of the study to allow for adaptation to the study diet. Starting on study Day 8, participants’ energy intake was reduced by ~30% of total energy needs for 28 days. At the conclusion of weight maintenance (Day 7) and energy restriction (Day 35), net acid extraction (NAE), nitrogen balance (NBAL), and ammonia and urea turnover were assessed from urine to determine the effects of energy restriction and KHCO_3_ supplementation.

### 2.3. Anthropometrics

Height was measured to the nearest 0.1 cm in duplicate using a wall mounted stadiometer. Body mass was measured to the nearest 0.1 kg using a calibrated digital scale (Seca, Los Angeles, CA, USA). Body mass was measured twice a week during weight maintenance and energy restriction to monitor study diet compliance and track weight loss.

### 2.4. Study Diet

All food and beverages, except water, were provided to participants for the entirety of this controlled feeding study. Meals were provided in 3-day menu cycles and prepared in the Human Nutrition Research Center on Aging (HNRCA) metabolic kitchen. Each meal was prepared in advance and checked for accuracy by study staff. Energy needs were individualized to each participant, using the Harris Benedict equation with a fixed factor of 1.2 to account for dietary thermogenesis and daily living activities. Dietary protein was provided at 1.0 g∙kg^−1^∙day^−1^ for both groups, during both weight maintenance and energy restriction phases of the study. Dietary protein was tightly controlled as it is a potent anabolic stimulus that has been shown to be effective in maintaining nitrogen balance [18]. An amount of 1.0 g∙kg^−1^∙day^−1^ was chosen to be consistent with recent recommendations from the PROT-AGE Study Group, that older individuals’ protein requirements are above the current recommended dietary allowance (RDA)of 0.8 g∙kg^−1^∙day^−1^ and should consume at least 1.0 g∙kg^−1^∙day^−1^ [19]. Dietary fat accounted for ≤30% of total energy at both phases and carbohydrate provided the remainder of the prescribed energy. Subjects were provided with a daily multivitamin/mineral supplement. The potential renal acid load (PRAL) of the study diets were determined as [20]:

PRAL = (Phos × 0.0366) + (Pro × 0.4888) − (Pot × 0.0205) − (Cal × 0.0125) − (Mag × 0.0263)

where Phos is phosphorus (mg∙day^−1^), Pro is protein (g∙day^−1^), Pot is potassium (mg∙day^−1^), Cal is calcium (mg∙day^−1^), and Mag is magnesium (mg∙day^−1^).

### 2.5. KHCO_3_ Supplement

Supplements were dispensed as 90 mmol∙day^−1^ KHCO_3_ in 7 gelatin capsules per day (12.86 mmol KHCO_3_ per capsule; Life Enhancement Products, Minden, NV, USA). The placebo (PLA) group received an equal number of capsules containing microcrystalline cellulose (Life Enhancement Products). Participants were blinded to which group they were assigned, but study staff were not. Consumption of KHCO_3_ or PLA capsules was initiated at the beginning of the energy restriction phase (Day 8). To ensure tolerance, participants gradually increased pill intake, consuming 3 capsules per day starting on study Day 8 and progress to 7 capsules per day by Day 10. Participants were instructed to take capsules with an 8-oz glass of water immediately after meals. A safety blood draw was conducted on Day 21 to ensure circulating potassium concentrations were within the normal range.

### 2.6. Net Acid Excretion

All urine measurements (NAE, NBAL, and ammonia and urea turnover) were determined from a single pooled 24-h urine. Urine collections were initiated at the conclusion of weight maintenance (Day 6) and energy restriction (Day 34) phases. Net acid excretion was measured using a modification of the Jorgensen titration method [21], as described by Chan [22] (NAE = titratable acid + NH_4_^+^ − HCO_3_^−^). Briefly, titratable acid—HCO_3_^−^ was assessed after addition of HCl, boiling the sample, and then titrating the sample to neutral pH. To measure the NH_4_^+^, formol was added to the sample to release H^+^ from NH_4_^+^ and the sample was again titrated to neutral pH. All titrations were carried out with a TIM 900 Titration Manager (Radiometer Analytical, Loveland, CO, USA).

### 2.7. Nitrogen Balance

Total nitrogen content of the urine was determined from a single pooled 24-h urine sample using pyrochemiluminescence. Apparent nitrogen balance was calculated as the difference of nitrogen intake minus urinary nitrogen excretion plus miscellaneous (estimated at 5 mg/kg) and fecal (estimated at 2 g/day) losses [23]. Total 24-h urinary nitrogen excretion was measured with a model FP-2000 nitrogen/protein determinator (LECO, St. Joseph, MI, USA).

### 2.8. Whole-Body Ammonia and Urea Turnover

Ammonia and urea turnover was assessed on Days 6 and 34 using the ‘End-Product’ method [24]. Both end-products, ammonia and urea, were assessed in the present study, as both can be influenced by alterations in the acid-base balance and weight loss [25,26,27]. Stable isotope, ^15^N-glycine, was administered in the morning following a 12-h overnight fast. Immediately before each isotope dosing, participants provided a baseline urine sample for determination of background ^15^N-ammonia and ^15^N-urea and then instructed to completely empty their bladders. A single oral dose of ^15^N-glycine (300 mg; Cambridge Isotope Laboratories, Andover, MA, USA) was dissolved in local tap water and consumed by the participant [28]. Urine was then collected for 24 h after dosing, ending with the first-void urine upon waking the following day. Urinary ammonia and urea excretion during the 24-h study were determined using Colorimetric Spectroscopy assays. The ^15^N enrichment of urinary ammonia and urea (ratio of tracer to tracee, *t*:*t*) was determined using isotope ratio mass spectroscopy (Metabolic Solutions, Nashua, NH, USA). The *t*:*t* ratio for the cumulative sample was corrected for the background ^15^N-ammonia and ^15^N-urea enrichment. Nitrogen intake was determined from analysis of 24-h consumption of provided foods and beverages. Ammonia and urea flux (Q), protein synthesis (PS), protein breakdown (PB), and net protein balance (NET) were calculated as:

Q (g N∙kg^−1^∙day^−1^) = [D/(corrected *t:t*)/24 h × body weight]

PS (g∙kg^−1^∙day^−1^) = [Q − (E/24 h × body weight)] × 6.25

PB (g∙kg^−1^∙day^−1^) = [Q − (I/24 h × body weight)] × 6.25

NET (g∙kg^−1^∙day^−1^) = PS − PB

where D denotes the oral dose of ^15^N (D = g glycine × 0.1972), corrected *t*:*t* specific for ammonia or urea enrichments, E denotes ammonia or urea excretion, and I denotes nitrogen intake.

### 2.9. Statistical Analysis

Mixed model repeated measured ANOVA was used to assess main effects of phase (weight maintenance vs. energy restriction), group (KHCO_3_ vs. PLA), and their interactions (phase-by-group) for body mass, energy and macronutrient intake, PRAL, NAE, urinary NH_4_^+^ concentration, NBAL, and ammonia and urea flux, synthesis, breakdown, and net balance. Unstructured covariance, placing no fixed pattern on the variance of the dataset, was determined as the appropriate model based on the Akaike’s information criteria. Bonferroni corrections for pairwise comparisons were performed if significant interactions were observed. Pearson’s correlation coefficient was used to examine associations between NAE to urinary NH_4_^+^ concentration, NBAL and ammonia and urea turnover following energy restriction. Data presented as mean ± standard deviation (SD). The α level for significance was set *p* < 0.05. Data were analyzed using IBM SPSS Statistics for Windows Version 22.0 (IBM Corp., Armonk, NY, USA).

## 3. Results

### 3.1. Participants, Body Mass, Pill Compliance, and Dietary Intake

A total of 17 individuals enrolled to participate in this randomized placebo-controlled trial. One participant withdrew due to gastrointestinal discomfort related to the study pill (KHCO_3_) and was excluded from the analysis. So, a total of 16 individuals completed data collection. There was no significant difference in age between KHCO_3_ (63 ± 4 years) and PLA (65 ± 3 years). During weight maintenance there was no statistically significant difference in body mass or BMI by group (Table 1). Following 28 days of energy restriction, all participants lost significant (*p* < 0.05) body mass and reduced their BMI, with no significant differences between groups.

Adherence to consuming study capsules was high, with no significant difference between KHCO_3_ (98 ± 3%) and PLA (97 ± 2%). There was no significant difference between groups in energy and macronutrient intake during weight maintenance (Table 2). Per protocol, total energy, carbohydrate, and fat intakes were lower (*p* < 0.05) during energy restriction compared to weight maintenance, with no significant differences between groups. The PRAL of diet was greater (*p* < 0.05) during the energy restriction phase compared to that during the weight maintenance phase, independent of group.

### 3.2. Net Acid Excretion and Nitrogen Balance

**Figure 1:**
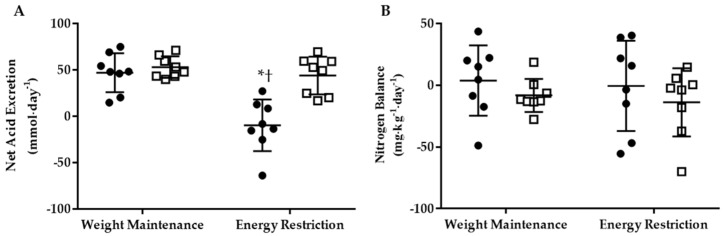
The 24-h net acid excretion (**A**) and nitrogen balance (**B**) for KHCO_3_ (●) and PLA (□) at Weight Maintenance and Energy Restriction. * Different than Weight Maintenance; *p* < 0.05. † Different than PLA; *p* < 0.05.

A phase-by-group interaction (*p* < 0.05) was observed for 24-h NAE, which significantly declined within the KHCO_3_ group pre- vs. post energy restriction, and was lower than PLA (Figure 1A). There was no difference in 24-h urinary NH_4_^+^ concentrations between phase or group (KHCO_3_ Weight Maintenance: 20.8 ± 5.0 mmol∙day^−1^, Energy Restriction: 16.2 ± 13.8 mmol∙day^−1^; PLA Weight Maintenance: 23.2 ± 3.8 mmol∙day^−1^, Energy Restriction: 23.1 ± 6.5 mmol∙day^−1^). However, when one outlier was removed from KHCO_3_ following energy restriction, a phase-by-group interaction (*p* < 0.05) was observed with NH_4_^+^ in KHCO_3_ (11.4 ± 6.5 mmol∙day^−1^) being lower than PLA (23.1 ± 6.5 mmol∙day^−1^). With the outlier removed, 24-h NH_4_^+^ was associated (*r*^2^ = 0.786; *p* < 0.05) with NAE. However, no statistically significant effect of phase or group was observed for 24-h NBAL (Figure 1B).

### 3.3. Whole-Body Ammonia and Urea Turnover

#### 3.3.1. KHCO_3_ vs. PLA

**Figure 2:**
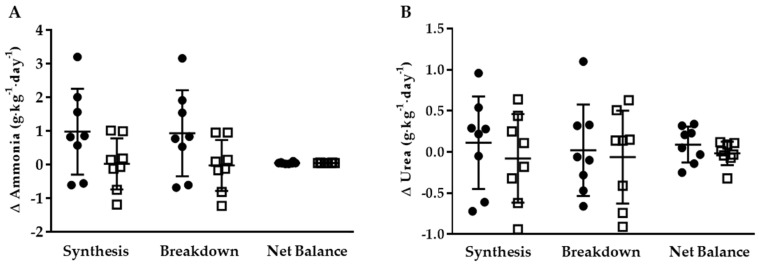
Delta 24-h whole-body ammonia (**A**) and urea (**B**) synthesis, breakdown, and net balance for KHCO_3_ (●) and PLA (□).

At the end of the weight maintenance run-in period, there were no significant differences in whole-body ammonia or urea synthesis, breakdown, or net balance in the two groups (Table 3). Furthermore, ammonia or urea synthesis, breakdown, or net balance did not differ significantly in the two groups following the energy restriction phase. There was no significant difference between groups for change in whole-body ammonia or urea synthesis, breakdown, or net balance (Figure 2). Though not statistically different, the change in ammonia synthesis and breakdown was numerically higher (*p* = 0.09) in KHCO_3_ compared to PLA following energy restriction.

#### 3.3.2. Energy Restriction

Overall, whole-body ammonia net balance was higher (*p* < 0.05) at the end of the energy restriction phase compared to the end of the weight maintenance phase in both groups (Table 3). No other significant energy restriction effect was observed for whole-body ammonia synthesis and breakdown or whole-body urea synthesis, breakdown, or net balance.

**Figure 3:**
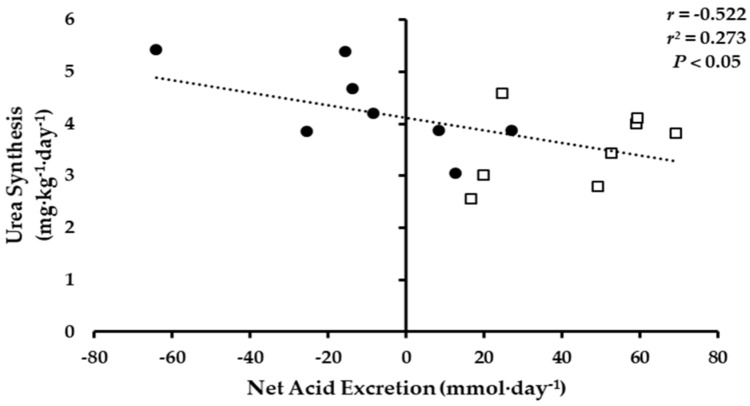
Association of 24-h Net Acid Excretion to urea whole-body urea synthesis, KHCO_3_ (●) and PLA (□).

The 24-h NAE was inversely associated with whole-body urea synthesis (Figure 3; *p* < 0.05). No significant associations were observed between 24-h NAE and NBAL or ammonia and urea turnover.

## 4. Discussion

This pilot study demonstrated that, following 28 days of 30% energy restriction, 90 mmol·day^−1^ of KHCO_3_ resulted in reductions in NAE compared to PLA. Reductions in NAE occurred despite PRAL being slightly higher in the energy-restricted phase; due to the maintenance of protein intake between phases, suggesting this difference did not impact the results of the study. Additionally, when one outlier was removed, 24-h urinary NH_4_^+^ concentrations were lower following energy restriction in KHCO_3_ versus PLA. Despite differences in in NAE and NH_4_^+^, there were no statistically significant differences between KHCO_3_ and PLA supplementation on NBAL, or whole-body ammonia and urea synthesis, breakdown, and net balance. The KHCO_3_ group had numerically higher whole-body ammonia synthesis and breakdown compared to PLA following energy restriction, although the difference did not meet statistical significance. Additionally, with the groups combined, NAE was inversely associated with whole-body urea synthesis. This finding suggests that reducing NAE with an alkaline supplementation may have some benefit on whole-body protein synthesis as the ‘End-Product’ method assumes that urinary enrichments reflect the amino acid pool being taken up into proteins [29,30].

With aging, there is an increase in the steady-state extracellular H^+^ and reductions in HCO_3_^−^ [10], likely due to alterations in kidney function, limiting the adaptive mechanisms responsible for maintaining acid-base homeostasis [31]. It is well documented that acidosis is detrimental to protein metabolism, promoting negative protein balance due to blunting of protein synthesis [11,32] and elevating whole-body protein breakdown [33]. Alterations in protein metabolism can likely be attributed to compensatory mechanisms which attenuate declines in blood pH through increased skeletal muscle degradation to donate nitrogen to reduce acidosis by accepting H^+^ ions and then be excreted in the urine [25,34]. This increase in muscle proteolysis, while beneficial in terms of maintaining blood pH, may compromise skeletal muscle integrity, as increased urinary nitrogen excretion leads to a net negative nitrogen balance, a marker of muscle loss. Although previous human [14,15,16,17] and animal [34] studies have reported benefit of KHCO_3_ to reduce urinary nitrogen excretion, this pilot study observed no statistical difference in NBAL, or ammonia and urea turnover when consumption of KHCO_3_ occurred during 28 days of 30% energy restriction. While energy restriction can increase urinary nitrogen excretion, resulting in a larger negative nitrogen balance [35,36], potentially masking the effects of KHCO_3_, this did not appear to be case based on data in the present study. Nevertheless, in this small pilot, KHCO_3_ supplementation did not significantly affect NBAL after energy restriction compared to PLA.

After energy restriction, change in whole-body ammonia synthesis and breakdown was numerically higher in the KHCO_3_ group (*p* = 0.91) compared to PLA, but not statistically different. It is difficult to determine if these numerically higher values indicate some benefit of KHCO_3_, as higher rates of both protein synthesis and breakdown would not result in an increase in net protein balance. However, the inverse association observed between NAE and whole-body urea synthesis would suggest that lower NAE is indicative of higher whole-body protein synthesis. It is important to note that while both ammonia and urea enrichments are derived from the metabolic nitrogen pool, there are differences in nitrogen contributions to both end-products, which result in differences in rates of turnover [37]. Urinary ammonia is primarily derived from glutamine, suggesting that ammonia enrichments are more reflective a muscle, as glutamine is primarily produced in the muscle [38,39]. Since urea is produced in the liver, its enrichment is considered to be more reflective of splanchnic protein turnover [38,39]. Discrepancies in the end-product pools in the present study may indicate that KHCO_3_ supplementation increases rates of both muscle protein synthesis and breakdown (i.e., turnover) compared to PLA, while lower NAE potentially results in higher rates of splanchnic protein synthesis. Though whole-body ammonia and urea enrichments offer an indirect assessment of muscle protein turnover, future investigations should consider using isotope methodologies that directly estimate rates of skeletal muscle protein synthesis. Additionally, longer investigation should be conducted to determine the effects of KHCO_3_ supplementation on changes in body composition following weight loss.

Findings from the current study may suggest KHCO_3_ supplementation alters whole-body protein synthesis; however, the current pilot study was underpowered to observe differences between groups. Based on a mean between-group difference of 0.96 mg·kg^−1^·day^−1^ and a common standard deviation of 0.98 mg·kg^−1^·day^−1^ for whole-body ammonia synthesis, with α set at 0.05 and β at 0.20, a sample size of 16 participants per group would be required to observe a statistical difference [40]. To achieve statistical significance for whole-body urea synthesis with a mean between-group difference of 0.091 mg·kg^−1^·day^−1^ and common standard deviation of 0.16 mg·kg^−1^·day^−1^, would have required 49 participants per group. In a recent publication by Ceglia and Dawson-Hughes [17], it was reported that with a sample size of 79 and 74 consuming a placebo versus 1.5 mmol∙kg^−1^∙day^−1^ (~110 mmol∙day^−1^) KHCO_3_, respectively, nitrogen balance (urinary nitrogen excretion relative to nitrogen intake) was increased in participants consuming KHCO_3_ supplementation. Findings from this past investigation suggests that KHCO_3_ supplementation may be beneficial in improving nitrogen retention and that a larger sample size than the current study is required to observe statistically significant differences.

## 5. Conclusions

The present pilot study observed that 90 mmol∙day^−1^ KHCO_3_ was effective in reducing 24-h NAE following 28 days of 30% energy deficit. Differences in NAE did not result in between group differences in NBAL or ammonia and urea turnover. However, an inverse association was observed between NAE and whole-body urea synthesis at the end of the energy restriction phase of the study. This data may indicate that KHCO_3_ supplementation has some benefit on improving whole-body urea synthesis; however, the sample size in this pilot study was too small to observe statistical differences. Future investigations may require a sample size of 49 per group to achieve sufficient power.

## Figures and Tables

**Table 1:** Body Mass and Body Mass Index ^1^.

	Weight Maintenance		Energy Restriction		Delta	
	KHCO_3_	PLA	KHCO_3_	PLA	KHCO_3_	PLA
Body Mass (g)	89.9 ± 6.8	87.7 ± 10.5	85.1 ± 7.4 *	83.8 ± 10.0 *	−4.8 ± 1.6	−3.9 ± 0.9
BMI (kg·m^−2^)	28.9 ± 1.3	28.0 ± 2.5	27.3 ± 1.6 *	26.8 ± 2.4 *	−1.6 ± 0.5	−1.2 ± 0.3

PLA, placebo; BMI, body-mass index. ^1^ Data mean ± SD. * Different than Weight Maintenance; *p* < 0.05.

**Table 2:** Energy and macronutrient intake ^1^.

	Weight Maintenance		Energy Restriction	
	KHCO_3_	PLA	KHCO_3_	PLA
Energy (kcal·day^−1^)	2642 ± 244	2580 ± 296	1818 ± 231 *	1802 ± 189 *
Protein (g·day^−1^)	90 ± 7	89 ± 10	89 ± 7	88 ± 10
Carbohydrate (g·day^−1^)	409 ± 42	398 ± 47	257 ± 44 *	257 ± 26 *
Fat (g·day^−1^)	78 ± 7	76 ± 8	51 ± 6 *	50 ± 5 *
PRAL (mEq·day^−1^) ^2^	25 ± 5	24 ± 6	27 ± 5 *	26 ± 5 *

^1^ Data mean ± SD. ^2^ PRAL; potential renal acid load. * Different than Weight Maintenance; *p* < 0.05.

**Table 3:** Whole-body ammonia and urea turnover ^1^.

	Weight Maintenance		Energy Restriction	
	KHCO_3_	PLA	KHCO_3_	PLA
Ammonia (g∙kg^−1^∙day^−1^)				
Synthesis	3.78 ± 1.00	3.46 ± 0.91	4.76 ± 1.20	3.48 ± 1.25
Breakdown	2.79 ± 1.00	2.48 ± 0.91	3.72 ± 1.21	2.45 ± 1.25
Net Balance	0.99 ± 0.22	0.98 ± 0.09	1.04 ± 0.03 *	1.03 ± 0.12 *
Urea (g∙kg^−1^∙day^−1^)				
Synthesis	4.17 ± 0.98	3.61 ± 0.55	4.28 ± 0.82	3.53 ± 0.71
Breakdown	3.91 ± 0.90	3.25 ± 0.54	3.93 ± 0.79	3.19 ± 0.75
Net Balance	0.26 ± 0.19	0.36 ± 0.8	0.35 ± 0.20	0.34 ± 0.14

^1^ Data mean ± SD. * Different than Weight Maintenance; *p* < 0.05.
